# Enhancing the electrocatalytic oxygen reduction activity of PrFeO_3_ through B-site manganese substitution

**DOI:** 10.1039/d6ra04148e

**Published:** 2026-08-17

**Authors:** P. Parida, B. B. Nayak, A. R. Panda, T. Hati, S. Samanta, P. Parhi

**Affiliations:** a Department of Chemistry, Ravenshaw University Cuttack Odisha-753003 India pparhi@ravenshawuniversity.ac.in +91-8895193144; b Technical Physics Division, Bhabha Atomic Research Centre Mumbai Maharashtra 400085 India

## Abstract

The advancement of effective rare-earth-dominated perovskites as electrocatalysts for the Oxygen Reduction Reaction (ORR) holds significant relevance for energy storage systems, such as fuel cells. In this study, we present a simple method to improve the electrocatalytic performance of PrFe_1−*x*_Mn_*x*_O_3_ (*x* = 0.1, 0.2, and 0.3), referred to as PFMO-0.1, PFMO-0.2, and PFMO-0.3, respectively, which are produced using the sol–gel technique. The structural characteristics and morphology of the synthesized materials were thoroughly investigated using XRD, FESEM, TEM, EDS, and XPS. The material characterization indicates that PFMO-0.1 possesses a greater particle size and an increased number of oxygen vacancies, which are advantageous for ORR. The results were verified by testing the electrocatalytic activity using a rotating disk electrode (RDE) and a rotating ring disk electrode (RRDE) in 0.1 M KOH. PFMO-0.1 exhibited the highest onset potential of 0.76 V *vs.* RHE, a notable limiting current of −3.64 mA cm^−2^, and a superb half-wave potential *E*_1/2_ = 0.59 V *vs.* RHE. The electron transfer number (*n*), calculated from a RRDE, is nearly equal to 4. The PFMO-0.1 catalyst's superior catalytic performance is further supported by its diminished Tafel slope, which indicates faster reaction kinetics for the ORR. Furthermore, chronoamperometric studies validated that the PFMO-0.1 electrocatalyst demonstrates greater longevity and improved methanol-resistant performance in relation to the standard commercial Pt/C catalyst.

## Abstract 2

This study illustrates how Mn substitution at the B-site improves the catalytic performance of a PrFeO_3_ perovskite for the oxygen reduction reaction (ORR).

## Graphical abstract

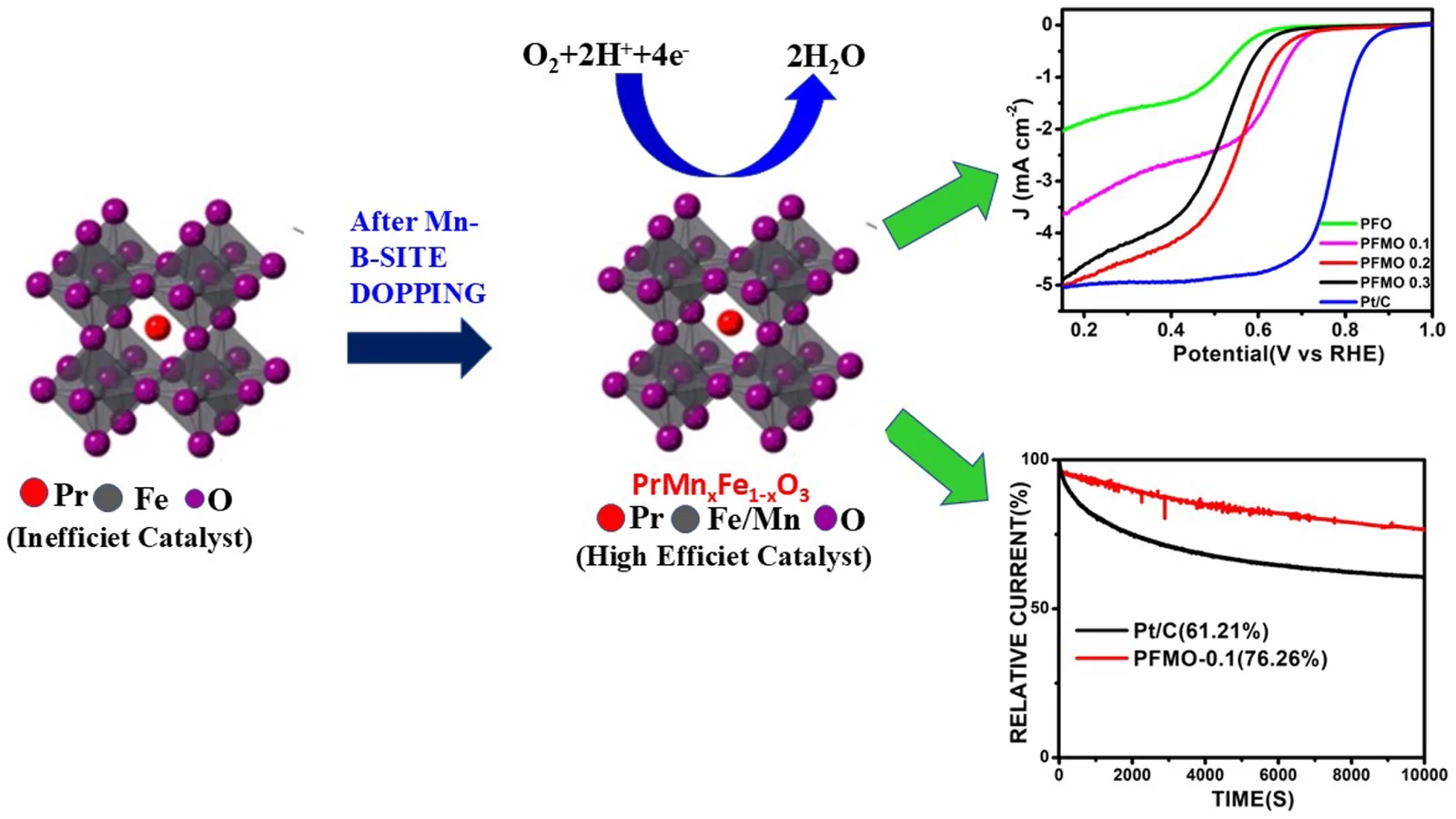

## Introduction

With the rapid advancement of worldwide industrial growth and urban development, the reliance on fossil fuels is intensifying, resulting in greater demand and contributing to environmental contamination and the greenhouse effect.^1,2^ The solution to these challenges lies in a range of technologies that support energy storage and conversion. Consequently, researchers worldwide are seeking practical solutions to satisfy energy requirements by focusing on technologies such as energy converters, photovoltaic modules, rechargeable lithium batteries, and regenerative metallic–air electrochemical cells,^3–6^ with fuel cells having attracted significant interest in recent years.^7^

In the realm of fuel cell advancements, Direct Methanol Fuel Cells (DMFCs) have captured significant interest in recent years.^8–10^ These electrochemical devices generate electricity directly from the chemical energy found in methanol and oxygen, making them a promising avenue for sustainable and clean energy solutions. However, the cathodic kinetics, *i.e.*, ORR, is the half of the fuel cell, tends to be comparatively sluggish in DMFCs. The slow-moving dynamics of ORR at the cathode arise from the considerable irreversibility of the reaction. Therefore, the creation of an efficient electrocatalyst is vital for their successful commercialization.^11^ For this purpose, platinum-based catalysts are superior, but their high cost, limited availability, and inadequate durability pose significant problems. Consequently, current research focuses on developing durable, economically viable, and rare metal catalysts.^12–16^ Although cost–efficient transition metal oxides have shown promise as ORR catalysts, they have established a superior record as ORR catalysts.^17–19^

Recently, rare-earth-based perovskites have been suggested as a substitute for noble-metal-based electrocatalysts in their use for ORR. In perovskite materials characterized by the chemical formula ABO_3−*δ*_, rare-earth cations reside at the eight vertices of a cubic lattice (A site) while a transition metal (B-site) inhabits the central cavity, coordinated octahedrally by six oxygen atoms.^20^ In a perovskite structure, the massive A-site cations are generally bigger than the B-site cations. The smaller B-site cation is typically coordinated by six oxygen anions, forming an octahedral shape. The structural stabilities and the resulting characteristics are predominantly influenced by the A-site ion and the B-site ion, respectively.^21^

Perovskite oxides, such as the LnMnO_3_ (where Ln is a rare earth element) family, are considered promising catalytic materials for the oxygen cathode process because of their abundance, impressive catalytic efficacy for oxygen, and economic advantages over noble-metal catalysts.^22,23^ Specifically, lanthanum-based compounds, like LaMnO_3_, exhibit superior electrocatalytic and ORR performance among the LnMnO_3_ series, following the sequence: La > Pr > Nd > Sm > Gd > Y > Dy > Yb.^36^ Substitution of ions at either the A-site or B-site of perovskite structures 
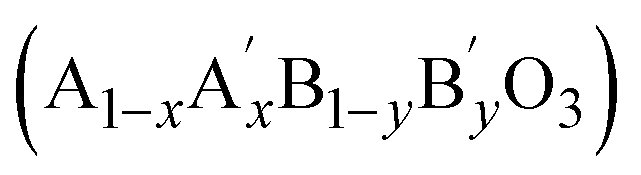
 is a common strategy for creating materials with superior ORR characteristics.^24^ The catalytic performance of mixed metal compounds is significantly influenced by the oxidation state of the group B elements of the B-site ion.^25^ Perovskite oxides, especially those exhibiting high B-site that form covalent bonds with oxygen atoms formed and an adsorbed cation e_g_ occupancy close to unity, are promising ORR electrocatalysts. The B-site group B metal ions, including Fe, Co, Ni, and Mn, serve as a viable active center for the ORR. It has been shown that partially replacing the B-site ion with a secondary transition metal enhances electrocatalytic performance, largely owing to the cooperative interaction connecting the two cations. Furthermore, the resulting compounds frequently exhibit modified structures, such as porous structures, which promote the transfer of oxygen or electrolytes to the perovskite material's surface, thereby boosting activity.^24^ Praseodymium ferrite (PFO) is a perovskite oxide characterized by an orthorhombic structure that has garnered considerable interest due to its diverse structural adaptability and multifunctional characteristics, such as mixed electronic and ionic conduction, as well as excellent thermal stability. Its distorted perovskite framework, influenced by the size of Pr^3+^ at the A-site and the octahedral FeO_6_ configuration at the B-site, facilitates a powerful interplay between the crystal lattice and its functional properties. When synthesized at the nanoscale, PFO displays heightened surface reactivity and enhanced defect transport routes, establishing it as a positive compound for application in catalysis, sensing, and energy conversion.^26^ PFO nanomaterials present a substantial opportunity to expand their functional capabilities. Substituting Pr at the A-site and Fe with Mn at the B-site enables systematic modulation of ionic radii, valence states, and local distortions while maintaining the perovskite structure. The resulting high-entropy PFMO nanomaterials enjoy improved phase stability, increased defect density, and synergistic cationic interactions that can considerably elevate catalytic performance and charge transport.

Few studies have investigated the ORR action of lanthanide perovskite systems based on lanthanum. However, recent research has explored the dual functional catalytic properties of Mn-doped LaFeO_3_ for both ORR and Oxygen Evolution Reaction (OER) activities in basic conditions. The finding indicates that the amount of Mn doping considerably influences the catalytic function. At *x* = 0.5, the catalyst LaFe_0.5_Mn_0.5_O_3_ (LFM) showcases the optimal performance. The limiting current density in a 0.1 mol L^−1^ KOH solution is 7 mA cm^−2^, significantly surpassing that of the commercial Pt/C catalyst (5.5 mA cm^−2^). Furthermore, the activity of the doped catalyst is also exceptional compared to that of the marketable Pt/C regarding extended stability. The remarkable catalytic efficiency of LFM can be attributed to its rich O^2−^/O^−^ species and reduced polarization resistance following the introduction of Mn impurities.^27^ Additionally, LaMn_*x*_Co_1−*x*_O_3_ (*x* = 0, 0.25) perovskites have been examined as innovative nanomaterial catalysts for the extraction of thiophene compounds from fuels *via* catalytic and ultrasound-enhanced chemical oxidation of sulfur.^28^

This manuscript details an investigation into the influence of the introduction of impurities at the B-site on the ORR performance of PrFe_1−*x*_Mn_*x*_O_3_ perovskite oxides, where x values of 0.1, 0.2, and 0.3 correspond to PFMO-0.1, PFMO-0.2, and PFMO-0.3, respectively. The materials were obtained using the sol–gel process. The study specifically explores the ORR catalytic performance of all synthesized PFMO perovskite oxides in 0.1 M KOH, including an analysis of their reaction kinetics and stability.

## Experimental section

### Material synthesis method

The PrFe_1−*x*_Mn_*x*_O_3_ (PFMO) powders, specifically PFMO-0.1, PFMO-0.2, and PFMO-0.3, high-purity Pr(NO_3_)_3_, Mn(ac)_2,_ and Fe (NO_3_)_3,_ served as the starting precursors for synthesis using an enhanced sol–gel technique, all acquired from Sigma-Aldrich. Each metal was individually dissolved in a precise stoichiometric ratio using 10 mL of deionized water. A round bottom flask containing 100 mL of water was inserted an water bath, after successive addition of the metal solutions and citric acid (CA), along with glycine, which serves as a chelating agent, was incorporated into the metal ion solutions, maintaining a molar ratio of CA : glycine : metal ions at 2 : 2 : 1. The solid–liquid blend was gently stirred to achieve the desired stoichiometric ratio for the PrFe_1−*x*_Mn_*x*_O_3_ compositions. The resulting slurries were then constantly agitated in a water bath at 80 °C till complete gel evolution occurred. The gels were subsequently dried at 250 °C for 30 minutes, and the final PFMO fine particles were produced by calcining the dried gels at 700 °C for 4 hours.

### Material characterisation

The crystal form of the synthesized perovskites was identified *via* X-ray diffraction (XRD) using a Rigaku Ultima IV diffractometer equipped with Cu-Kα radiation (*λ* = 1.54178 Å). Patterns were recorded covering a 2*θ* range of 20° to 80° at a scanning rate of 4° min^−1^. Surface structure was analyzed using a Zeiss Gemini SEM 300 field emission scanning electron microscope (FESEM), while internal microstructure and particle dimensions were estimated *via* an FEI Technai G2S-Twin transmission electron microscope (TEM). Furthermore, X-ray photoelectron spectroscopy (XPS) with a monochromatic Al-Kα source was employed to investigate the outermost layer composition and the oxidation states of the component elements.

### Electrochemical measurements

Electrochemical measurements were performed using a Metrohm Autolab 204 RDE workstation fitted out with a conventional triple-electrode configuration. A modified glassy carbon (GC) electrode was employed as the working electrode, while a graphite rod and an Hg/HgO electrode served as the counter electrode and reference electrode respectively. To prepare the working electrode, the catalyst ink was uniformly cast onto a pre-cleaned GC surface. This ink was formulated by dispersing 5 mg of the synthesized PFMO materials and 1 mg of carbon (Vulcan-XC 72) in a mixture of 1 mL of isopropanol and 1.5 mL of deionized water. Afterward, 25 µL of a 0.5% Nafion solution was mixed in, and the solution was agitated for 30 minutes. This ratio was chosen to strike a balance between intrinsic catalytic efficiency and electronic conductivity. An increased proportion of PFMO guarantees adequate exposure of perovskite-derived ORR active sites (Mn^3+^/Mn^4+^ redox centres and a surface rich in oxygen vacancies), while the additional carbon facilitates a robust conductive network, reduces polarization resistance, and enhances the distribution of the catalyst on the electrode. Ratios with diminished carbon content resulted in inferior film conductivity, while increased carbon proportion diluted the active phase and lowered the observed mass activity. Consequently, the 5 : 1 PFMO-to-carbon ratio presents an ideal balance, facilitating consistent kinetic assessment under RDE conditions while guaranteeing repeatable ORR performance.^29^ The working electrodes featured active surface areas of 0.07067 cm^2^ and 0.196 cm^2^ for RDE measurements and RRDE measurements, respectively; both were dried in a desiccator before use.

Cyclic Voltammetry (CV) and Linear Sweep Voltammetry (LSV) were conducted in a 0.1 M KOH solution, saturated with either N_2_ or O_2_ as required. The RDE measurements were performed at a scanning speed of 10 mV s^−1^ potential variation of 0.067 to 1.067 V *vs.* the Reversible Hydrogen Electrode (RHE).

To quantify the number of electrons transferred and the peroxide yield (% H_2_O_2_), RRDE measurements were executed at a rotation speed of 1600 RPM and a scanning speed of 10 mV s^−1^, with the ring potential held at 0.5 V. All recorded potentials were subsequently calibrated to the RHE scale employing the following eqn (1).^30,31^1
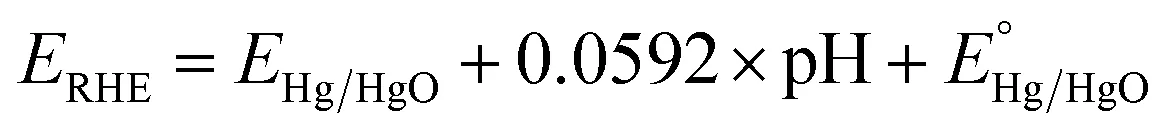
where 
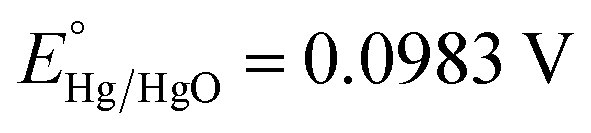
 and pH = 13.

During the assessment of a catalyst's efficiency for the oxygen reduction reaction, we focused on the consecutive aspect.

Onset potential (*E*_on_): the onset potential denotes the start of significant ORR activity and was determined as the potential at which a current density of −0.1 mA cm^−2^ was recorded.

Electron transfer number (*n*): serves as a key indicator of the reaction pathway. Ideally, an electrocatalyst should facilitate the direct 4-electron reduction of O_2_ to water H_2_O, avoiding the less efficient 2-electron pathway that produces hydrogen peroxide (H_2_O_2_).

Kinetic current (*J*_k_) and limiting current (*J*_L_): these metrics evaluate the reaction rate and peak speed.

Mass activity: this quantifies the current generated per unit mass of the catalyst, reflecting its mass-specific performance.

Electrochemical active surface area (ECSA): this metric determines the effective coverage of the catalyst that is accessible to the electrolyte and actively contributes to the electrochemical reaction.^32^

## Result and discussion

**Fig. 1:**
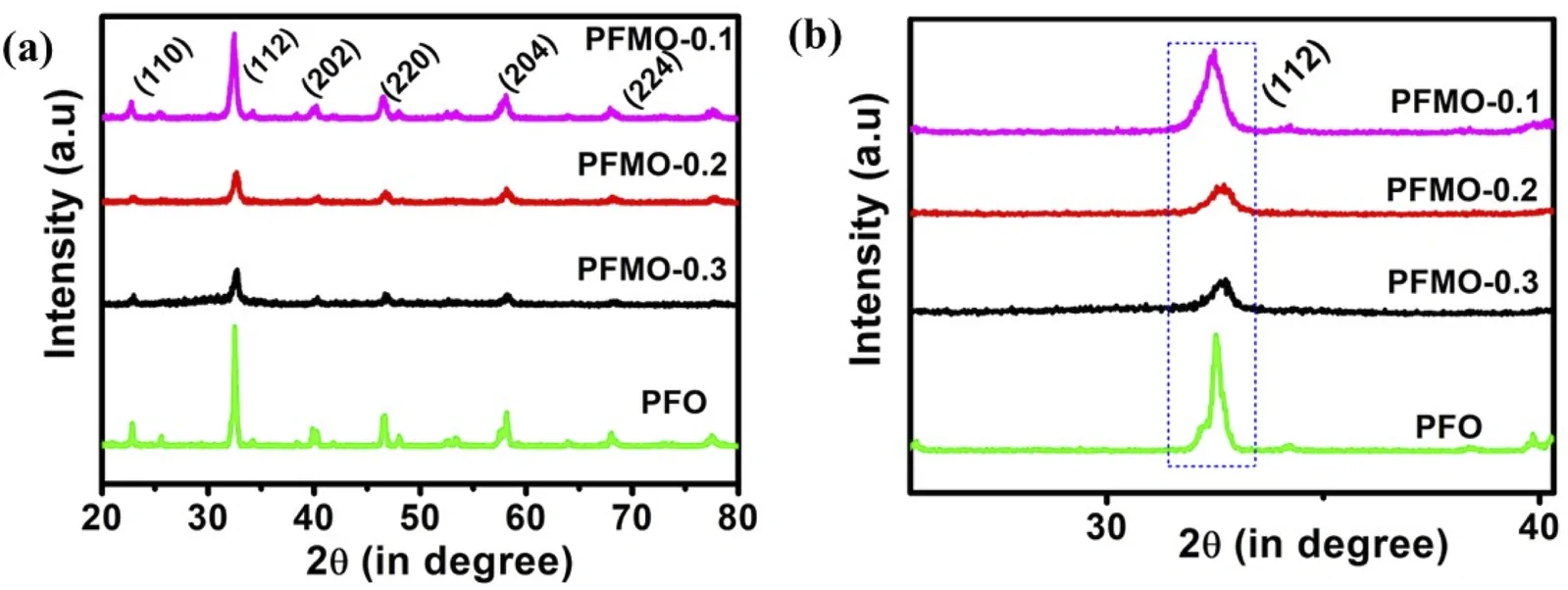
(a) X-ray diffraction spectra of different PrFe_1−*x*_Mn_*x*_O_3_ (b) magnified XRD spectra for (112) peak within the 2*θ* = 25°–40°ranges.

X-ray diffraction (XRD) analysis confirmed that all prepared PFMO and PFO perovskite oxides achieved a single-phase, orthorhombic structure after calcination at 700 °C for 4 hours, with no discernible impurities, indicating that the B-site Fe doping does not alter the fundamental crystalline framework. The main peaks correspond to the (110), (112), (202), (220), (204), and (224) crystal facets of PrFeO_3_ (ref. 33). As the manganese content increases, the XRD patterns, particularly the principal (112) peaks shown in the magnified view between 31° and 34° (Fig. 1(b)), exhibit a consistent shift towards higher 2*θ*. This systematic shift signifies a compression of the perovskite unit cell structure and a resultant lattice contraction. This contraction is primarily attributed to the compact ionic size of the use in place of Mn^4+^ (0.53 Å) compared to Mn^3+^(0.645 Å), a change governed by the discrepancies in the ionic size between the dopant and host ions.^34,35^

The crystallite size and *d*-spacing were determined from the (112) plane using the Scherrer equation.^36^2
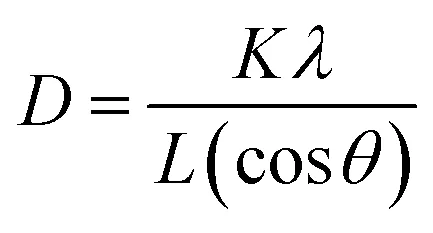
In the above equation, *D* is the crystal size, *K* is the shape factor (0.9), *λ* is the wavelength, *L* is the full width half maxima (FWHM), and *θ* is Bragg's angle.

The width is measured at the part of the topmost of the principal peak, but the base is truncated to disregard the tail of the shoulder. This method is the least accurate and is typically utilized for rapid assessments. If the shoulder is pronounced, applying the unmodified FWHM would result in a broadening error, producing a diminished *D* value in the Scherrer equation:

For PFO and PFMO-0.1 samples, the shoulder likely indicates a slight secondary phase or lattice strain Table 1. In the analysis, this is addressed by deconvolving the peaks to ensure the FWHM accurately portrays only the coherent scattering domain of the primary phase, thereby keeping the crystallite size calculation representative of the bulk material.

**Table 1:** Crystallite diameter and interplanner spacing of the perovskite catalysts

Catalysts	Crystallite size (nm)	*d*-Spacing(Å)
PFO	6.39	2.759
PFMO-0.1	4.14	2.758
PFMO-0.2	3.85	2.741
PFMO-0.3	3.78	2.739

The PFMO perovskite with the highest Fe content, PFMO-0.1, was found to have larger particle dimensions and a greater *d*-spacing compared to the other PFMO compositions. This observation is consistent with Bragg's Law, which dictates that as the *d*-value decreases due to a reduction in ionic radius, the location of the diffraction peaks must consequently shift toward higher angles. A systematic shift of diffraction peaks relative to pristine PFO is observed, attributed to the substitution of larger Mn^+4^ ions for Fe^3+^, leading to lattice expansion and local structural distortion. Peak broadening suggests reduced crystallite size and increased microstrain, which are favourable for exposing catalytically active surface sites.

**Fig. 2:**
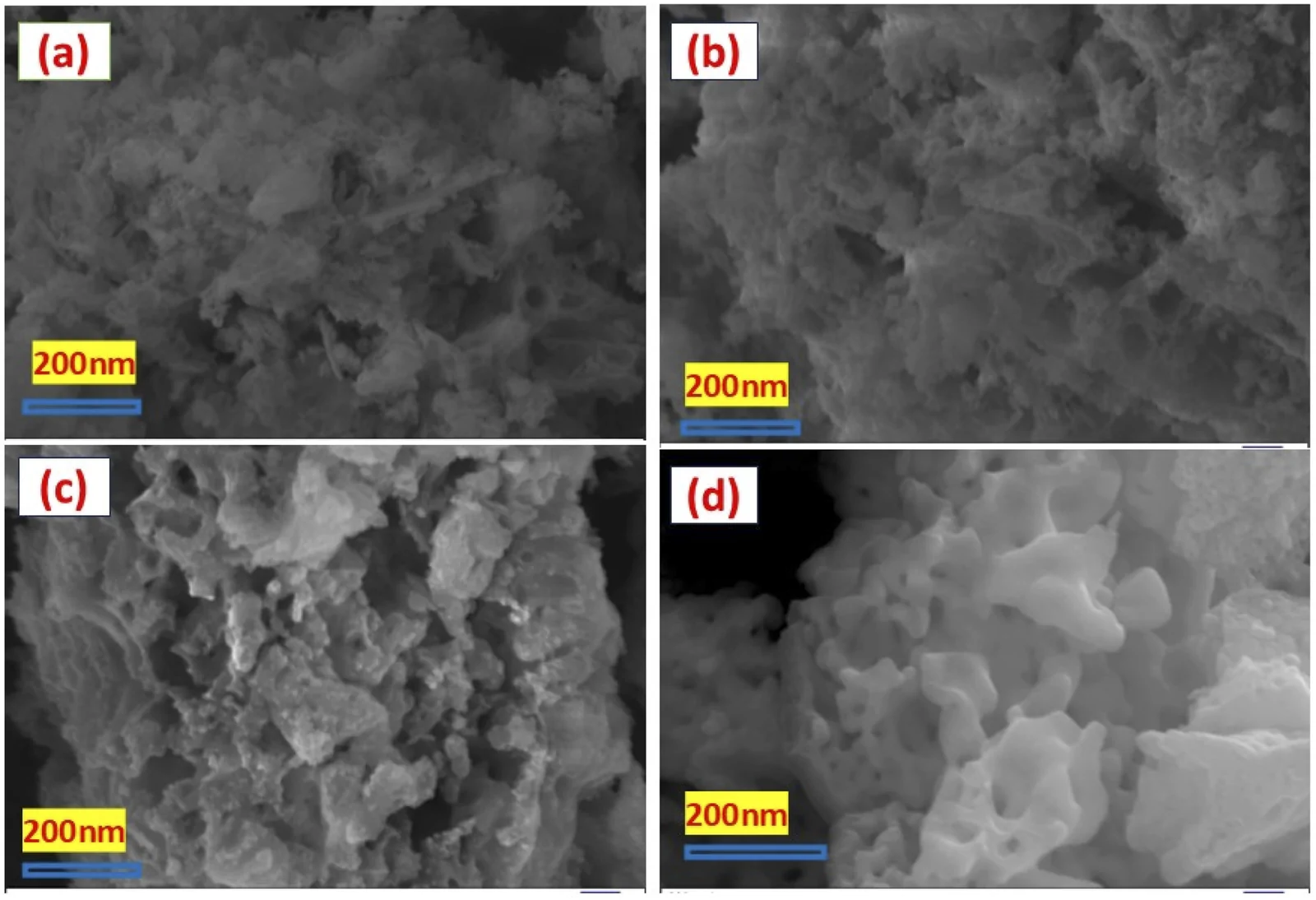
FESEM visuals of synthesized perovskites (a) PFMO-0.3, (b) PFMO-0.2, (c) PFMO-0.1, and (d) PFO at 200 nm.

The exploration of PrFe_1−*x*_Mn_*x*_O_3_ (*x* = 0.1, 0.2, 0.3) perovskite samples through scanning electron microscopy (SEM) demonstrated that Mn doping resulted in diminished particle aggregation Fig. 2.

Notably, the PFMO-0.1, distinguished by the least B-site Mn doping (*x* = 0.1), displayed the most significant particle aggregation clustering and the largest crystallite dimensions.

**Fig. 3:**
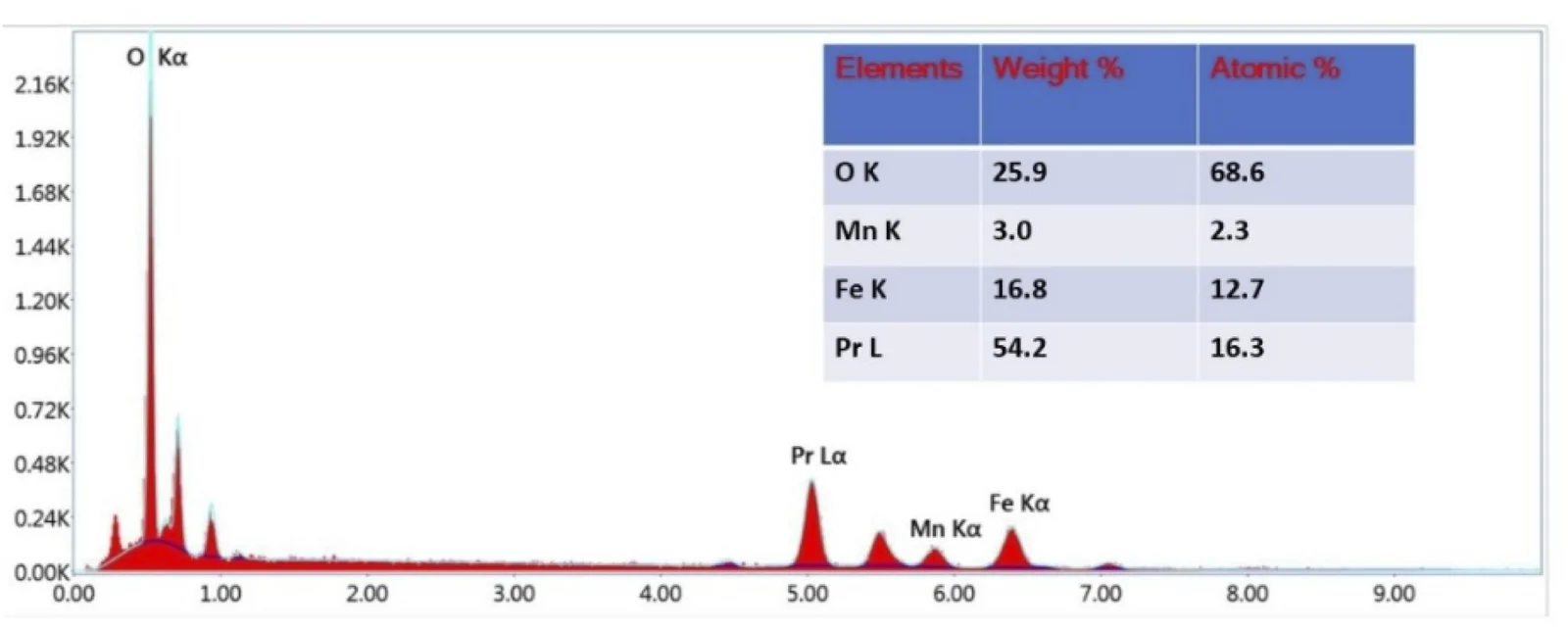
EDS elemental distribution content of PFMO-0.1.

Energy-dispersive spectroscopy (EDS) analysis showed that no other elements were present except Pr, Mn, Fe, and O. The ratios of these elements were very near their relative stoichiometric ratio, which indicates that the sample was well-crystallized with correct chemical composition Fig. 3.

**Fig. 4:**
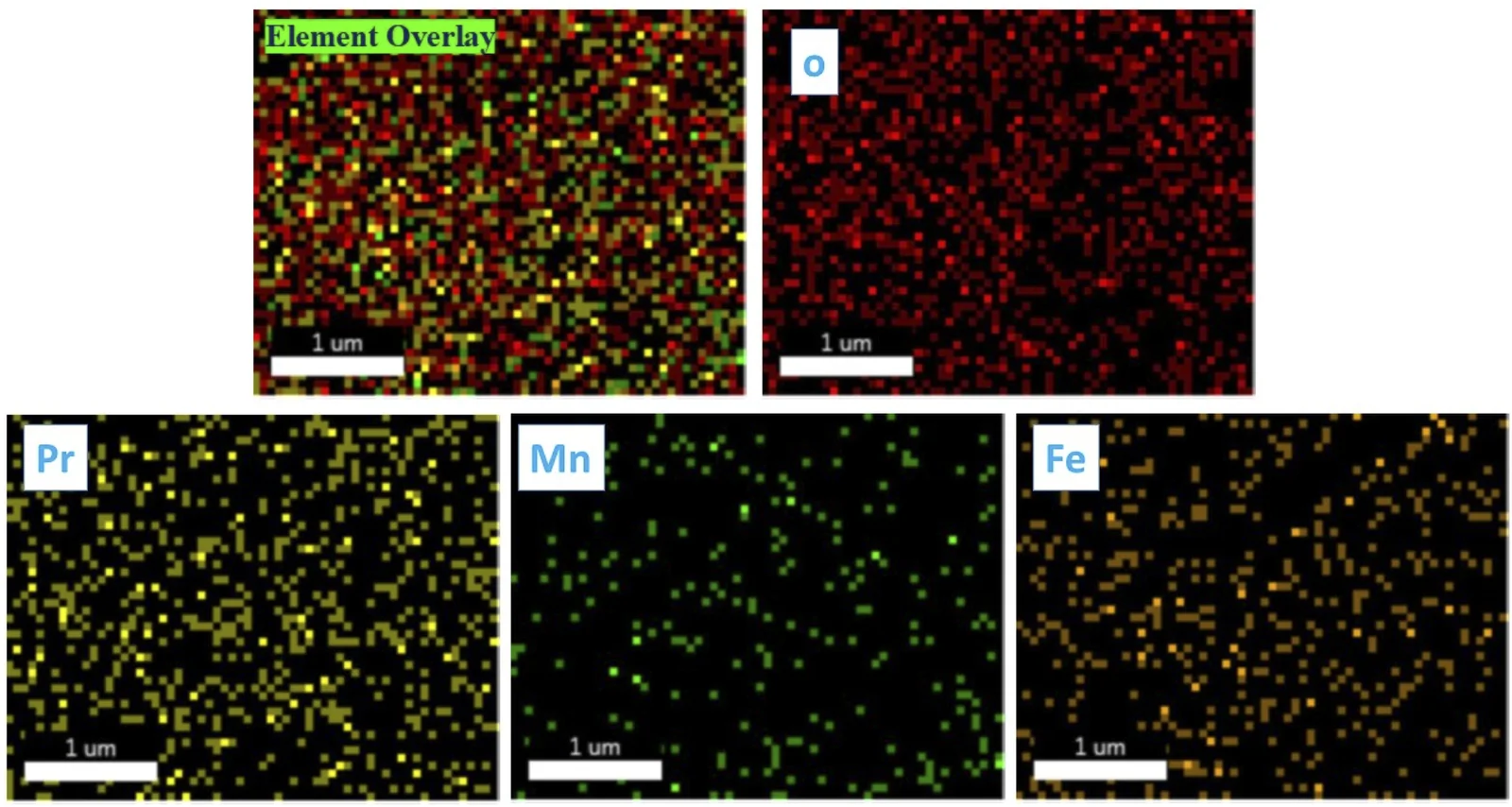
EDS elemental distribution mapping of PFMO-0.1.

Fig. 4, illustrating the EDS elemental mapping of the PFMO-0.1 sample in SEM mode, indicates a uniform distribution of every chemical element throughout the perovskite. A significant observation was the consistent spread of Mn, which suggested that the Mn atoms successfully substituted Fe at the B-site of the prepared sample.

**Fig. 5:**
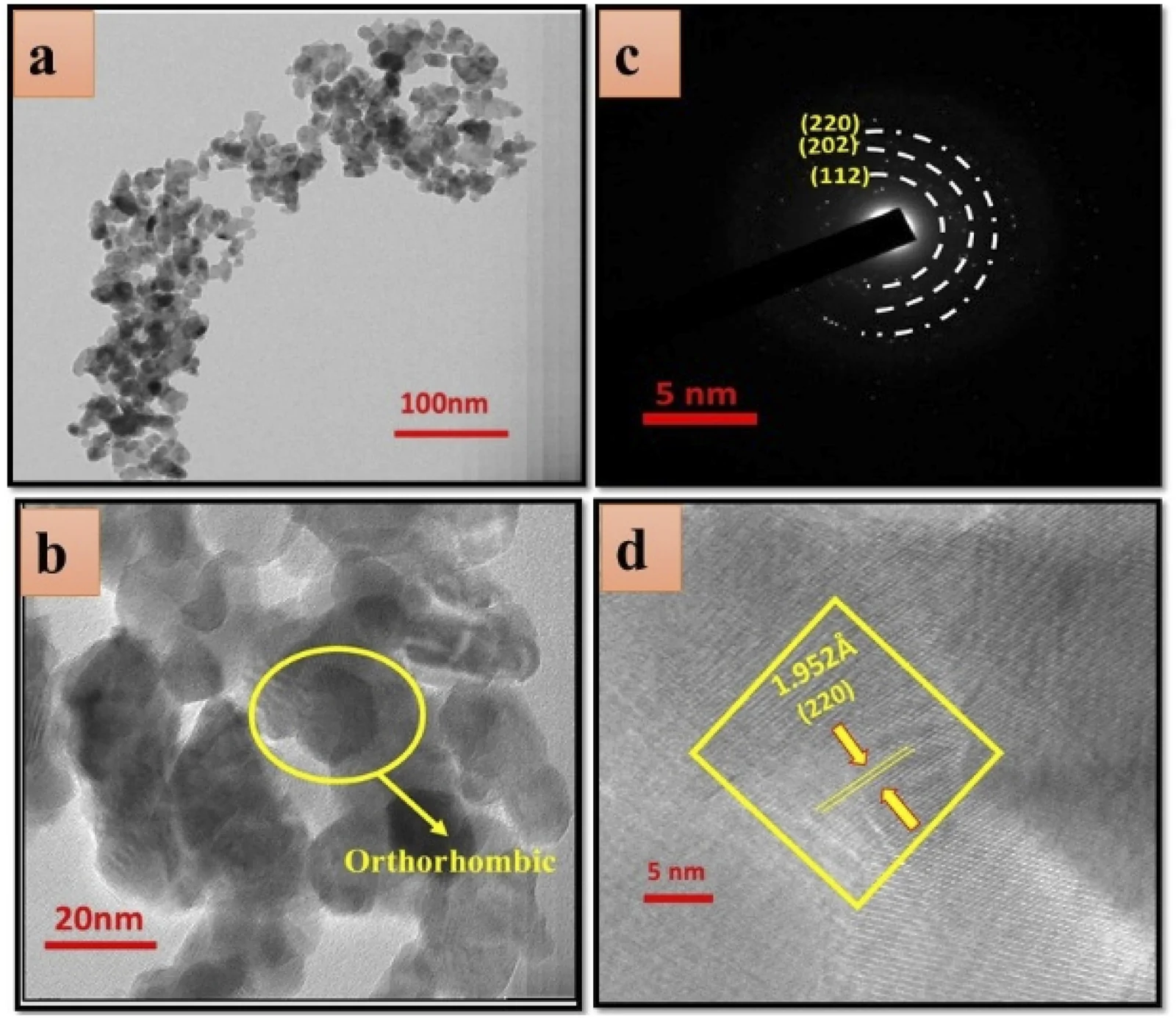
(a) Transmission electron microscopy (TEM) image (b), zoomed-in TEM view (c), selected area electron diffraction (SAED) design (d), HRTEM analysis of PFMO-0.1.

High-resolution transmission electron microscopy (HR-TEM) examination of the PFMO-0.1 perovskite (Fig. 5) revealed distinct lattice fringes, confirming its crystalline structure. Low-magnification TEM images (Fig. 5(a)) and a magnified view (Fig. 5(b)) clearly demonstrated an orthorhombic arrangement. The observed particle dimensions, ranging from 4 to 20 nm, were reconcilable with the X-ray Diffraction (XRD) results. Furthermore, the Selected Area Electron Diffraction (SAED) design (Fig. 5(c)) confirmed crystallinity, displaying three distinct rings (Fig. 5(c)) corresponding to the (112), (202), and (220) diffraction planes. The HR-TEM image (Fig. 5(d)) allowed the determination of the interplanar spacing for PFMO-0.1 as 1.952 Å, which is reconcilable with the (220) planes.^37^ High-resolution TEM (HRTEM) shows clear lattice fringes, confirming high crystallinity, while slight lattice distortion and discontinuities point to the presence of oxygen vacancies and defect-rich regions induced by Mn doping.

**Fig. 6:**
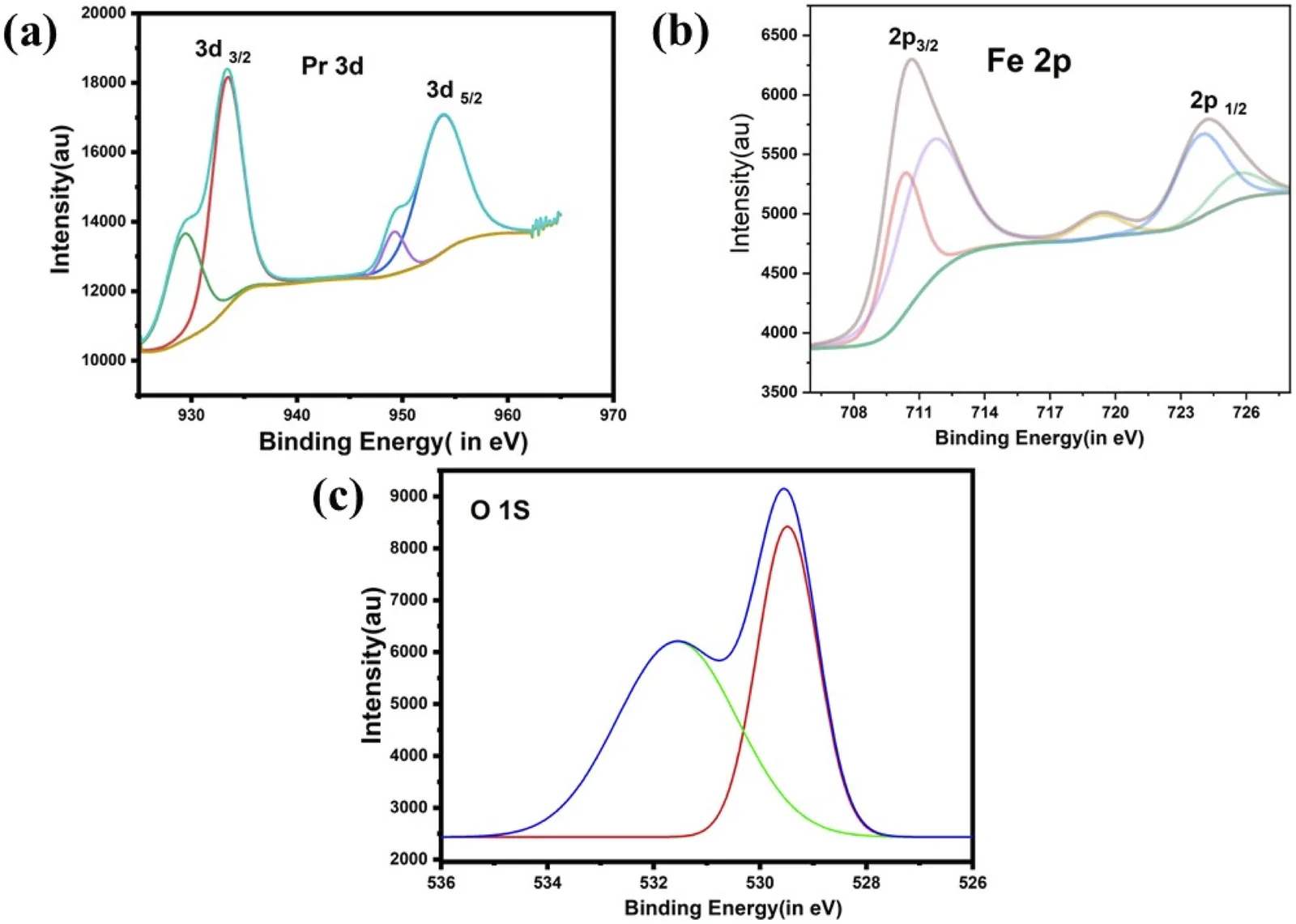
XPS spectra of (a) Pr 3d, (b) Fe 2p, and (c) O 1s for PFO perovskite.

X-ray photoelectron spectroscopy (XPS) was employed to investigate the chemical environment and oxidation states of the constituent elements in the sol–gel synthesized PFO sample. The deconvoluted XPS spectra of the O 1s, Fe 2p, and Pr 3d core levels are presented in Fig. 6. Different XPS peaks for O 1s are observed at around 529.34, 529.82 and 532.08 eV. The peak at approximately 529.4–529.9 eV is attributed to lattice oxygen associated with the Pr–O and Fe–O bonds in the PrFeO_3_ crystal structure, while the higher binding energy peak at 531.9–532.1 eV is assigned to surface-adsorbed oxygen species or oxygen-related defects.^38–42^ The Fe 2p spectrum exhibits two characteristic spin–orbit doublets with Fe 2P_3/2_ and Fe 2P_1/2_ peaks located at 710.85 eV and 724.23 eV, which correspond to +3 oxidation state, while those peaks at 710.3 eV (Fe 2P_3/2_) and 724 eV (Fe 2P_1/2_) are linked to +2 oxidation state, respectively.^43,44^ In Fig. 6(a) we observed the peak at 949.5 eV(3d_5/2_) and 929.5(3d_3/2_) corresponds to Pr^4+^. The peak for 953.6 eV for 3d_5/2_ and 933.3 eV for 3d_3/2_ can be linked to Pr^3+^.

**Fig. 7:**
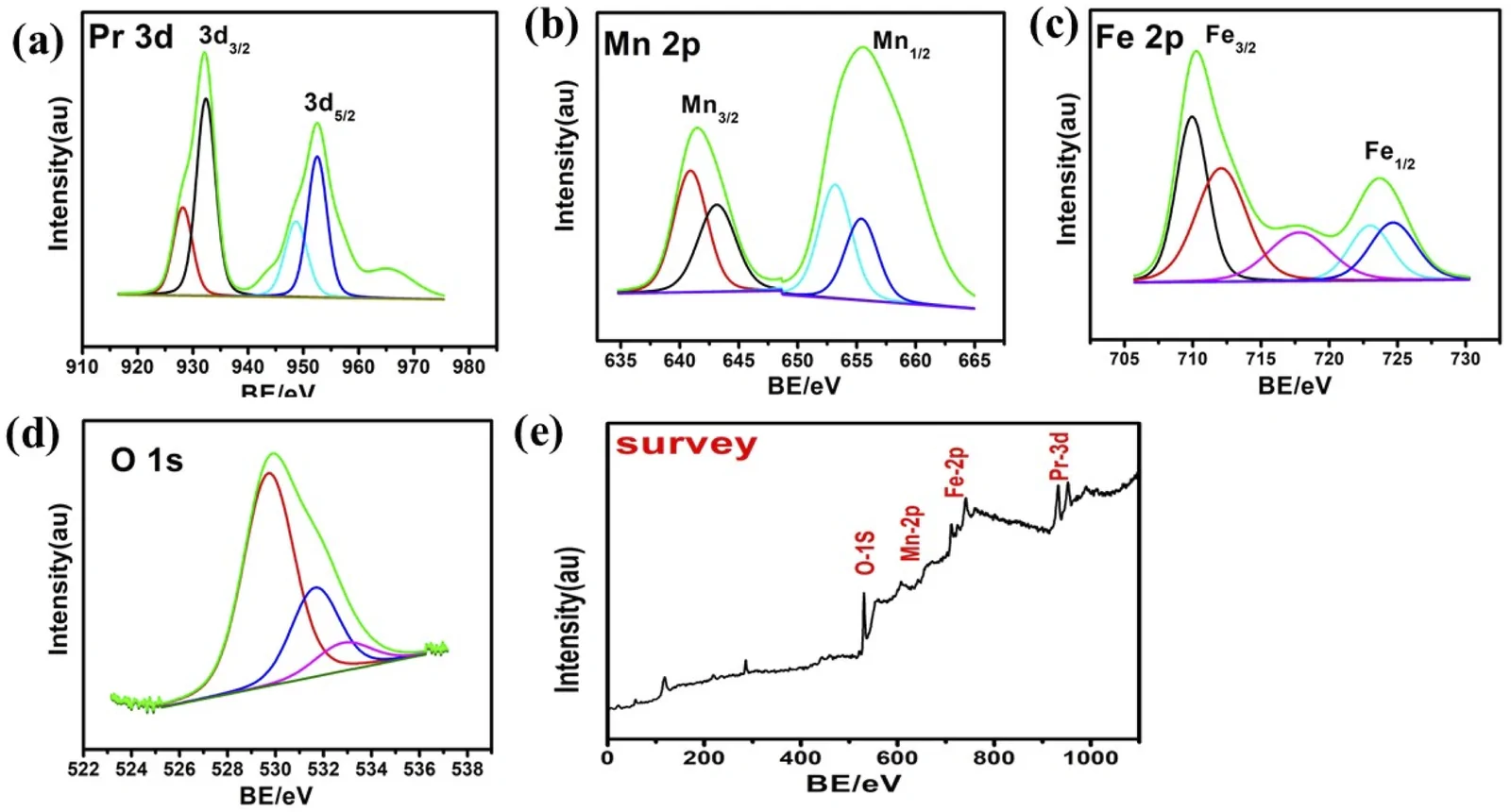
XPS spectra of (a) Pr 3d, (b) Mn 2p, (c) Fe 2p and (d) O 1s (e) survey for PFMO-0.1 perovskite.

Analysis of the PFMO-0.1 sample using XPS (Fig. 7) revealed its elemental makeup and oxidation states. Specifically, the Pr 3d spectra (Fig. 7(a)) displayed two major peaks at 932.52 eV and 952.98 eV, with respect to the Pr 3d_3/2_ and Pr 3d_5/2_ states.^45^ By identifying characteristic peaks of Pr^4+^ 3d_5/2_ and 3d_3/2,_ which are at 948.66 eV and 928.17 eV. The peak of 952.5 eV for 3d_5/2_ and 932.36 eV for 3d_3/2_ can be assigned to Pr^3+^.^46^ The data confirmed that the PFMO-0.1 sample exhibited a mixed oxidation state of (Pr^3+^/Pr^4+^). This Pr^3+^/Pr^4+^ redox couple is considered a significant factor influencing the electrocatalytic activity for the Oxygen Reduction Reaction (ORR) at the cathode surface.

The two peaks are found at 642.48 eV and 655.5 eV, respect to Mn 2p_3/2_ and Mn 2p_1/2,_ as seen in Fig. 7(b). The spin energy separation joining the Mn 2p_3/2_ and Mn 2p_1/2_ levels is 14.02, confirming the presence of Mn^+3^ (ref. 47). The peak located at 640.91 (Mn 2p_3/2_) and 653.18 eV (Mn 2p_1/2_) corresponds to Mn^+3^. Whereas the peaks located at 643.15 and 655.4 correspond to Mn^+4^ (ref. 41).

The Fe 2p spin–orbit doublets for the PFMO-0.1 sample were observed in the XPS spectrum (Fig. 7(c)) at 710.22 eV and 723.64 eV, corresponding to the Fe 2p_3/2_ and 2p_1/2_ states, with a resulting spin–orbit separation of 13.42 eV.^48^ Deconvolution of the spectrum revealed the presence of a mixed Fe oxidation state (Fe^3+^/Fe^2+^). Specifically, peaks attributed to Fe^3+^ were detected at 712.09 eV (Fe 2p_3/2_) and 724.68 eV (Fe 2p_1/2_), while those at 709.95 eV and 723 eV were linked to Fe^2+^.^49^ Furthermore, a satellite peak was observed for the sample at 717.81 eV.^50^

As shown in Fig. 7(d), the lower, intermediate, and higher binding energy peaks for O 1s observed at 529.69 eV (O I), 531.68 eV (O II), and 532.90 eV (OIII) are associated with lattice oxygen, adsorbed, and oxygen-deficient species.^51^ The existence of oxygen vacancies can also be clearly identified in the samples.^52^

From the XPS survey spectrum (Fig. 7(e)), the fundamental elements Pr, Fe, Mn, and O can be recognized in the prepared pure samples. Doping Pr -based samples with Mn at the B-site (*x* = 0.1) results in a slight shift in the oxygen peak in the XPS spectrum, suggesting the development of oxygen vacancies or lattice distortions, which may enhance functional characteristics like improved electrolytic performance.^53^ Shifts in host element core levels, *i.e.*, A, B-site cations, and O 1s indicate changes in bonding and electronic structure. Changes in the O 1s spectrum can indicate variations in oxygen vacancy concentration. XPS shifts in the transition metal and O 1s spectra can indicate stronger metal–oxygen covalent interactions.

Greater covalency generally means easier charge transfer between oxygen and the catalyst, more efficient oxygen activation. Many high-performance ORR perovskites exhibit enhanced metal–oxygen hybridization. In Table 2, the data presented indicate that an increased ratio of O_ads_/O_L_ corresponds to a higher concentration of oxygen vacancies.^54^

**Table 2:** Types of oxygen vacancies in the prepared catalyst

Catalyst	O_ads_ (XPS-O 1S)	O_L_ (XPS-O 1S)	O_ads_/O_L_
PFO	0.29	0.90	0.32
PFMO-0.1	0.256	0.6486	0.394

**Fig. 8:**
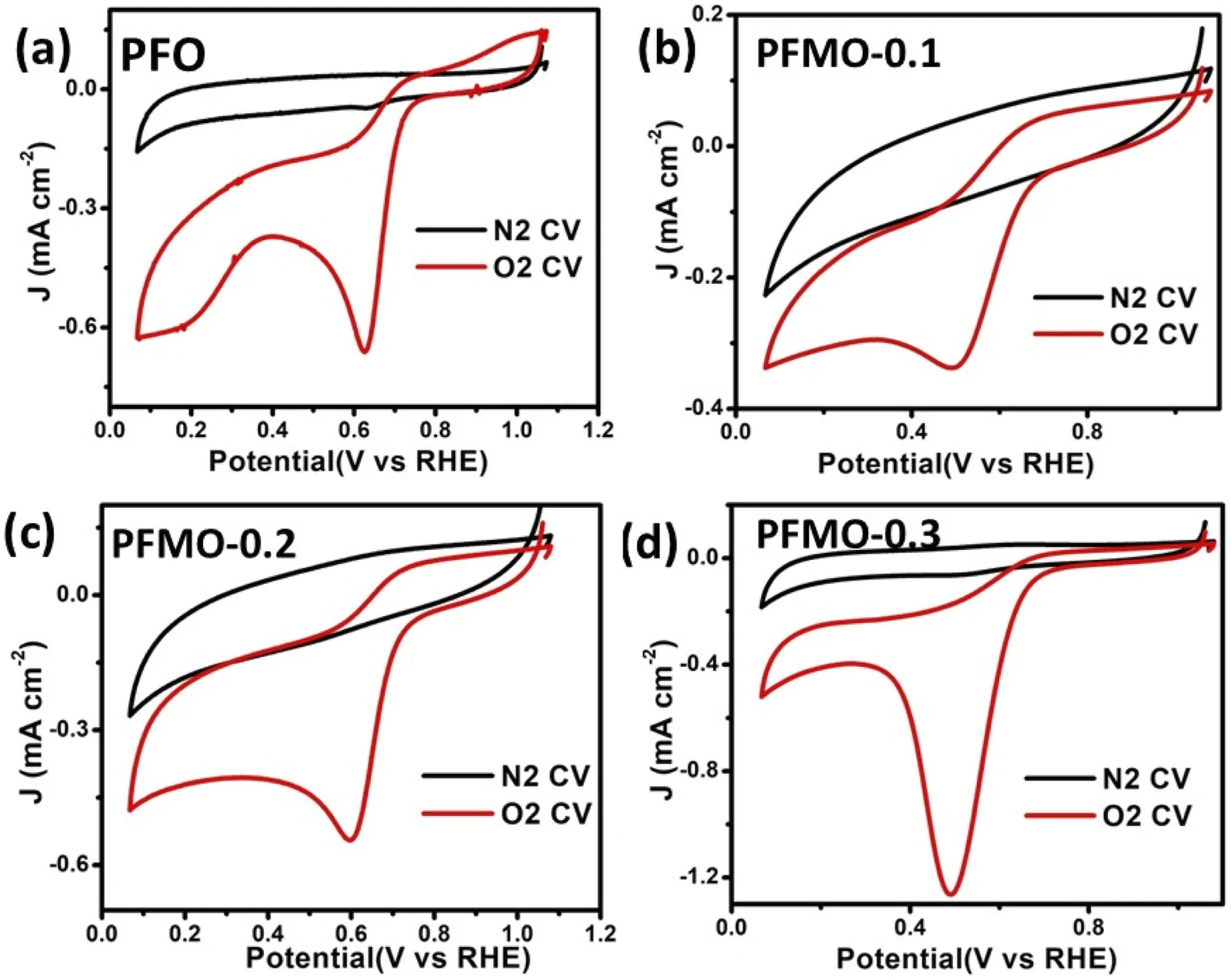
Cyclic voltammetry (CV) of all synthesised perovskite oxides by saturating N_2_ and O_2_ in a 0.1 M KOH solution at 10 mV s^−1^.

In CV experiments for all catalysts, spanning from 1.12 V to 0.12 V *vs.* RHE at a scan rate of 10 mV in 0.1 M KOH electrolyte, the observed electrochemical behavior confirmed their efficacy for the ORR.^55^ Specifically, when the electrolyte was nitrogen-saturated, no reduction peak was detected; however, a distinct cathodic peak appeared when the solution was oxygen-saturated (as shown in Fig. 8(a)–(d)). The distinctive oxygen-dependent reduction peak demonstrates the perovskite catalysts' efficacy in the ORR. The catalyst PFMO-0.1 among the synthesised ones showed highly desirable performance, with its reduction peak occurring at 0.76 V *vs.* RHE.

**Fig. 9:**
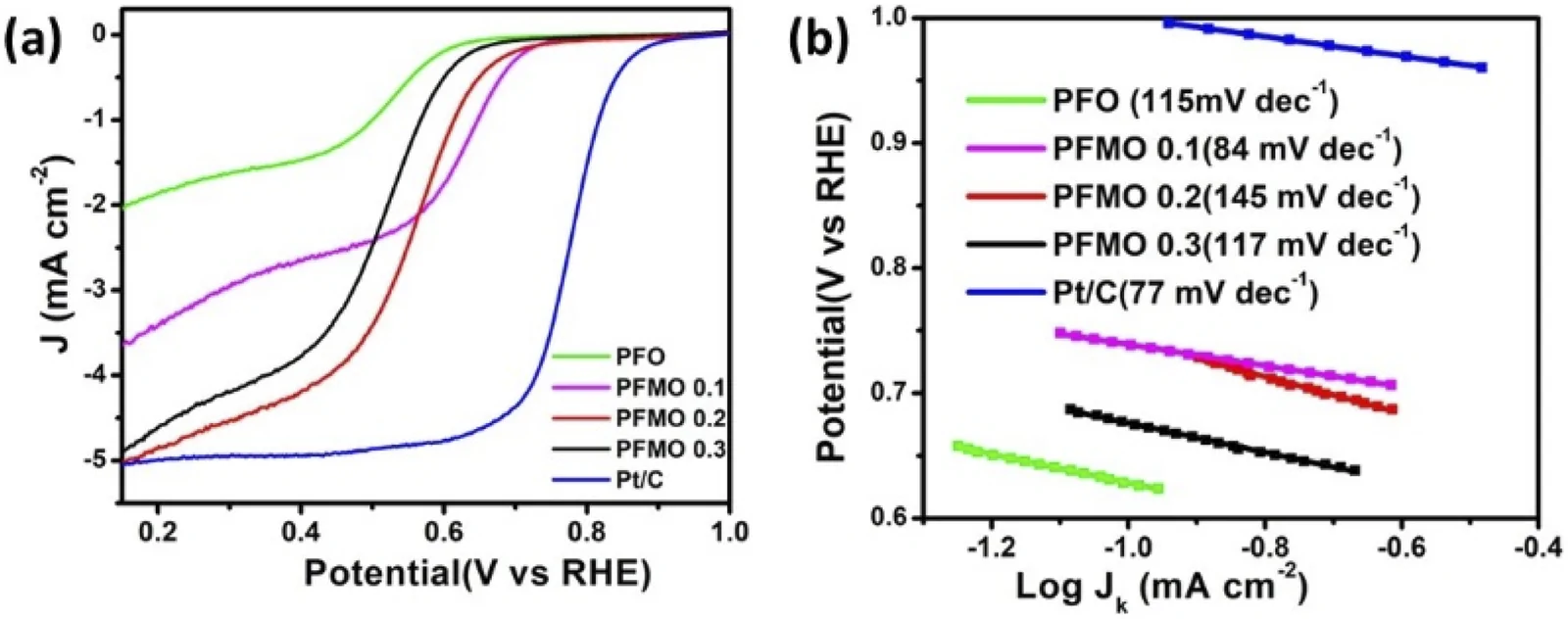
ORR performance of PrFe_1−*x*_Mn_*x*_O_3_ (*x* = 0.1, 0.2, 0.3) with different Mn content. (a) Linear sweep voltammograms (LSV) in O_2_-saturated 0.1 M KOH scanned at 10 mV s^−1^ at 1600 rpm. (b) Tafel curves of PFMO-0.1.

LSV curves of all the synthesized PFMO samples (*x* = 0.3, *x* = 0.2 and *x* = 0.1) were measured at a sweep rate of 10 mV s^−1^ in the potential range from 0.067 to 1.067 V *vs.* RHE. The measurements were taken at different rotation rates ranging from 400 rpm to 2500 rpm. As shown in Fig. 9(a), these LSV curves represent the ORR behaviour of the PFMO perovskite catalysts, with their performance compared to that of a commercial Pt/C catalyst, which was measured at 1600 rpm.^48^ The ORR s activity of PFMO perovskites was performed in an O_2_-saturated 0.1 M KOH solution with a three-electrode system connected to an RDE. The variation of the onset potential of PFMO perovskites because of different amounts of manganese doped at the B site of PFMO perovskite is shown in Fig. 9(a). It clearly indicates that with an increased amount of manganese doped at the B site, the onset potential of PFMO perovskite gradually decreases. The onset of the ORR is characterized by the potential at which a significant departure from the baseline current is observed; for the purposes of this study, this threshold is set at a current density of −0.1 mA cm^−2^.

The ORR onset potential of PFMO-0.3, PFMO-0.2, PFMO-0.1, and PFO are 0.70 V, 0.73 V, 0.76 V, and 0.65 V *vs.* RHE. The current densities of PFMO-0.3, PFMO-0.2, PFMO-0.1, and PFO were found at 1.12 V *vs.* RHE to be −4.8 mA cm^−2^, −5 mA cm^−2^, −3.64 mA cm^−2^, and −2.04 mA cm^−2^ as listed in Table 2. It is clearly noticed that PFMO-0.1 shows a higher positive onset potential among all the prepared catalysts.

The *J*_k_ is a crucial metric for assessing the catalytic performance of an ORR catalyst, which is estimated using a mass transport-corrected polarization curve and derived from the formula3
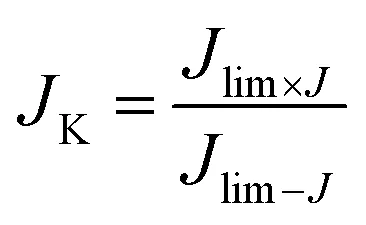
Here, *J*_lim_ and *J* are the limiting current, and the current is measured at a specific potential (0.83 V), respectively. To minimize mass transport-related errors, the materials were compared based on their *J*_k_ values at high potentials. The calculated *J*_k_ values for the PFMO samples were 0.051 for PFO, 0.018 for PFMO-0.3, 0.054 for PFMO-0.2, and a significantly higher 1.53 mA cm^−2^ for PFMO-0.1, which clearly surpasses those of the other electrocatalysts produced. The resulting kinetic current densities and corresponding onset potentials for all prepared perovskite catalysts are comprehensively summarized in Table 2.

The Koutecky–Levich (K–L) relationship was employed to evaluate the Tafel kinetics and electron transfer numbers (*n*), thereby elucidating the mechanistic pathways of the PFMO-based catalysts during the ORR.4
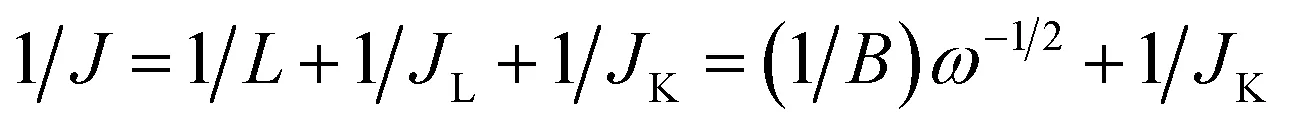
where, *B* = 0.62*nFC*_0_(*D*_0_)^2/3^*V*^1/6^.

The overall observed current density is *J*, while *J*_L_ and *J*_K_ represent the current densities formulated by diffusion and kinetic factors, respectively. Key parameters influencing the reaction include the angular velocity *ω*, the number of electrons transferred per O_2_ molecule (*n*), the Faraday constant (*F* = 96,485C mol^−1^), the O_2_ concentration in bulk (1.2 × 10^−3^ mol L^−1^), the O_2_ diffusion coefficient (*D*_0_ = 1.9 × 10^−5^ cm^2^ s^−1^), and the kinematic viscosity of the electrolyte (0.01 m^2^ s^−1^). The quantity of electron transfers (*n*) is crucial in the evaluation of the ORR feasibility, and its value can be calculated from the slope of the 1/*J vs. ω*^−1/2^, according to the K–L equation.

**Fig. 10:**
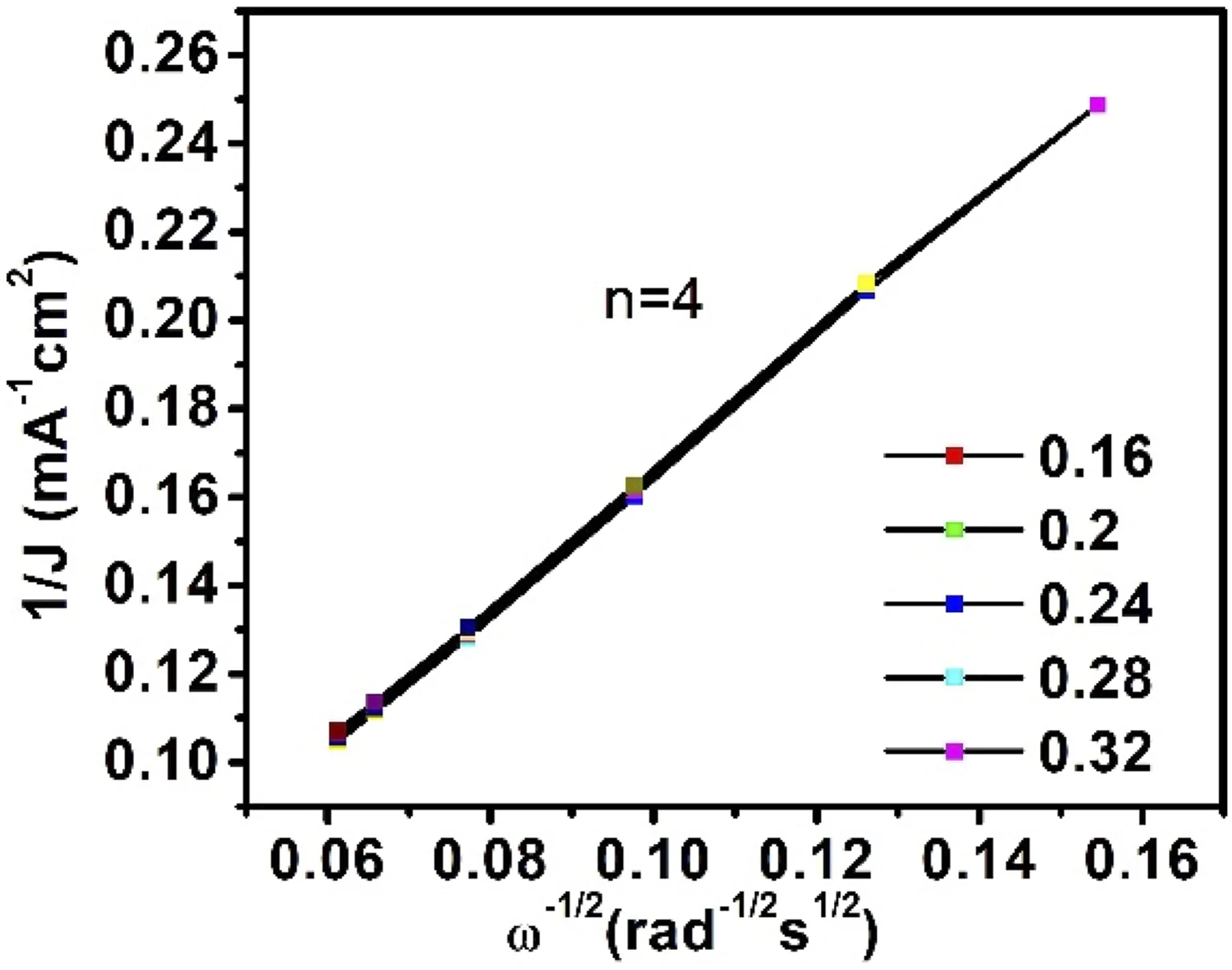
K–L plot at different electrode potentials for PFMO-0.1.

Tafel plots, acquired from the K–L relationship (eqn (4) and presented in Fig. 9(b)), elucidate the electrocatalytic kinetics of the prepared samples. As shown in Table 2, the Tafel slope for PFMO-0.1 is measured at 84 mV dec^−1^, which accounts for its lower Tafel slope among synthesized electrocatalysts and is highly comparable to that of commercial Pt/C (77 mV dec^−1^). Since a reduced Tafel slope indicates a quicker electron transfer during the reaction, the minimal slope observed for PFMO-0.1 suggests it possesses the highest ORR activity among the synthesized perovskites.^56^ The K–L diagram for PFMO-0.1 at varying electrode potentials is illustrated in Fig. 10. In this case, *J*^−1^ as a function of *ω*^−1/2^ is depicted for PFMO-0.1 across different electrode potentials. The slopes of the linear fits were used to calculate the number of electrons transferred (*n*) per O_2_ molecule. The parallel nature of the plots and the linearity of the plots suggest the ORR process for the PFMO-0.1 follows first-order kinetics with respect to dissolved O_2_ concentration (Table 3). In fact, the PFMO-0.1 catalyst showed a constant number of electrons transferred (*n* = 4) within the studied potential region.^57^

**Table 3:** Comparison of the electrochemical parameters of the synthesized perovskites

Catalysts	*E*_onset_ (V) *vs.* RHE (1600RPM)	*J*_L_ (mA cm^−2^)	*E*_1/2_ (V *vs.* Hg/HgO)	Tafel slope (mV dec^−1^)	Electron transport no. (*n*)	% Of HO_2_^−^	*J*_k_ (mA cm^−2^)	Mass activity (mA mg^−1^)	ECSA
**PFMO-0.1**	**0.76**	**−3.64**	**0.59**	**84**	**3.85**	**7.83**	**1.53**	**0.127**	**11.275**
**PFMO-0.2**	**0.73**	**−5**	**0.54**	**145**	**3.75**	**13.5**	**0.65**	**0.054**	**7.625**
**PFMO-0.3**	**0.70**	**−4.8**	**0.50**	**117**	**3.77**	**12.28**	**0.23**	**0.018**	**8.612**

**Fig. 11:**
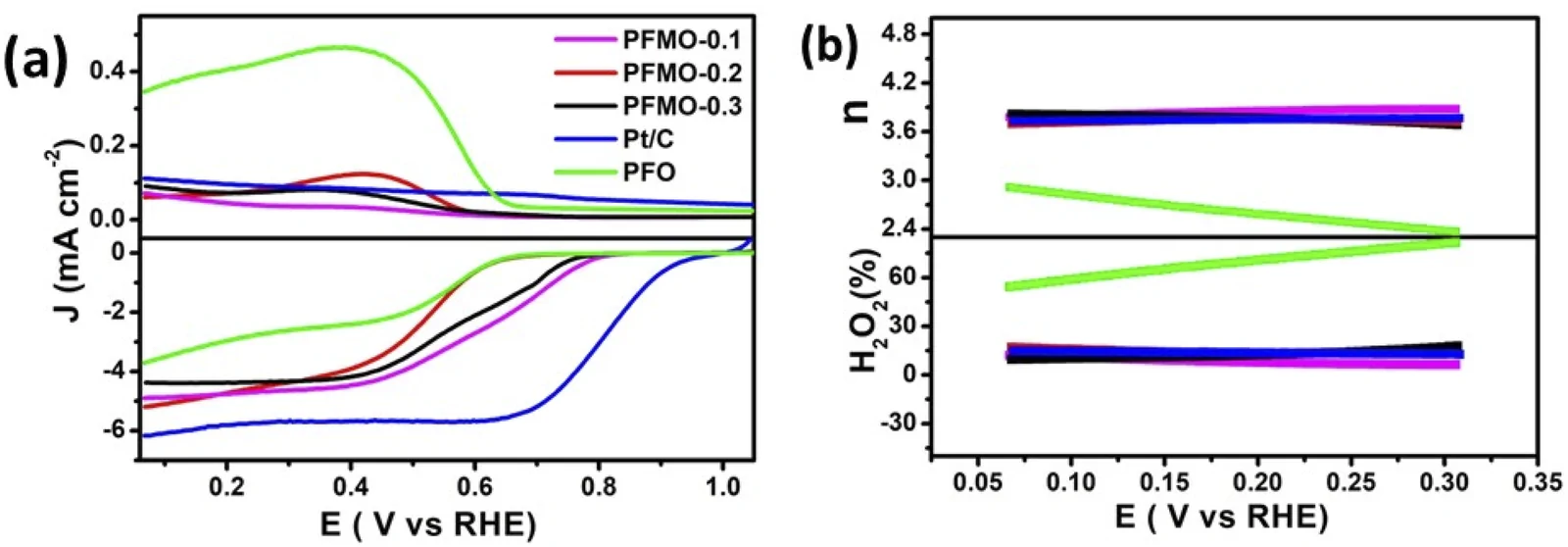
(a) RRDE polarisation graphs of PrFe_1−*x*_Mn_*x*_O_3_ (*x* = 0.1, 0.2, 0.3), PFO, and Pt/C nanoparticles at 1600 rpm in O_2_-saturated 0.1 M KOH solution. (b) Comparison of the electron transfer number and H_2_O_2_% of the prepared catalyst at various potentials.

Fig. 11(a) depicts the electrocatalytic activity of the prepared perovskite towards the ORR using the RRDE, where the *n* and the % of H_2_O_2_ produced during the ORR process were determined using the following equation5
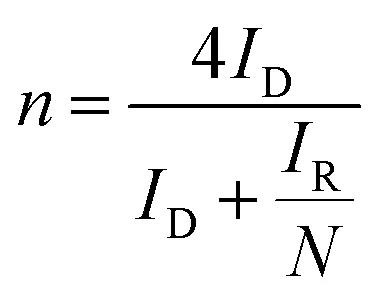
6
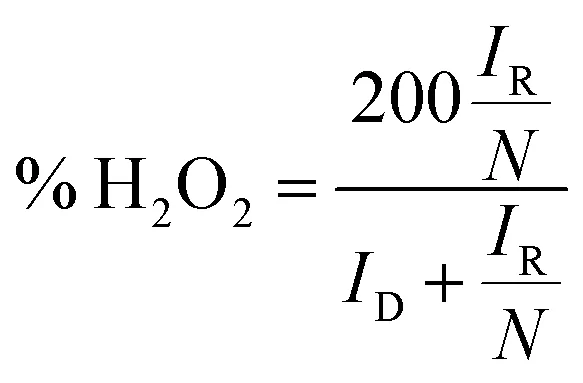


The number of electrons transferred (*n*) during the ORR process, calculated by the equation below where *I*_D_ is the current at the GC disk electrode, *I*_R_ is the current at the platinum ring electrode and *N* is the collection efficiency (*N* = 0.25, as determined by the RRDE manufacturer), was found to be 3.77 for PFMO-0.3, 3.75 for PFMO-0.2, and 3.85 for PFMO-0.1 (Table 2) at 0.2 V *vs.* RHE and for PFO (*n* = 2.90, % H_2_O_2_ = 52.17), Pt/C (*n* = 3.75, % H_2_O_2_ = 13.68). This data indicates that the ORR process predominantly follows a desirable four-electron pathway (*n* nearly equal to 4) with only minor formation of the undesired hydrogen peroxide byproduct Fig. 11(b), thereby confirming that PFMO-0.1 possesses the superior ORR catalytic activity among the synthesized materials.

**Fig. 12:**
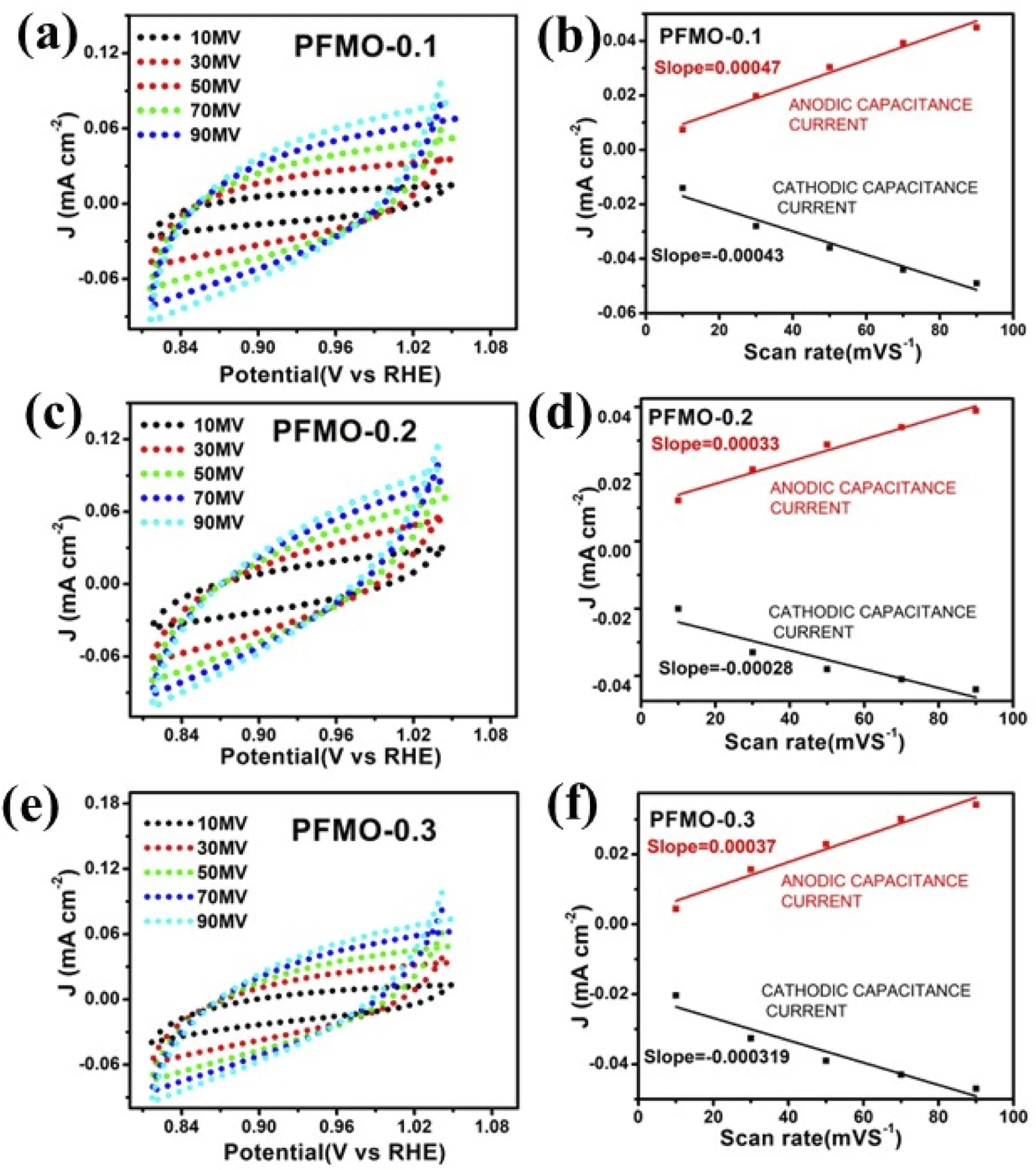
Cyclic voltammograms of (a) PFMO-0.1, (c) PFMO-0.2, (e) PFMO-0.3 at various scan rates (10–90 m s^−1^) in the non-faradic region in 0.1 mol L^−1^ KOH solution. (b) PFMO-0.1 (d), PFMO-0.2, and (f) PFMO-0.3 can be utilized for the evaluation of *C*_DL_.

Cyclic voltammetry is used for the determination of the electrochemical double-layer capacitance (*C*_DL_) for all synthesized PFMO perovskites by varying the scan rates from (10 ∼ 90 m s^−1^) given in Fig. 12, with the value *C*_DL_ calculated using a specific equation.

7 *j* = *vC*_DL_

The steady-state capacitive current *j* and the scanning speed *v* are denoted in the preceding eqn (7). The ECSA was determined by eqn (8) using the *C*_DL_ and the specific capacitance (*C*_S_), which is assumed to be 0.040 mF cm^−2^ for all the prepared perovskites.^58^

8 ECSA = *C*_DL_/*C*_S_

The ECSA values of PFMO-0.1, PFMO-0.2, and PFMO-0.3 in 0.1 M KOH are estimated as 11.275, 7.625, and 8.612 cm^2^, respectively. Among these prepared electrocatalysts, PFMO-0.1 has the largest active surface area. This larger surface area indicates enhanced ORR electroactivity.^59^

The PFMO-0.1 electrocatalyst was assessed for its reliability and resistance to crossover effects, crucial factors for its application in fuel cells or water electrolysis, examining its electrocatalytic selectivity toward methanol electrooxidation.^60^

**Fig. 13:**
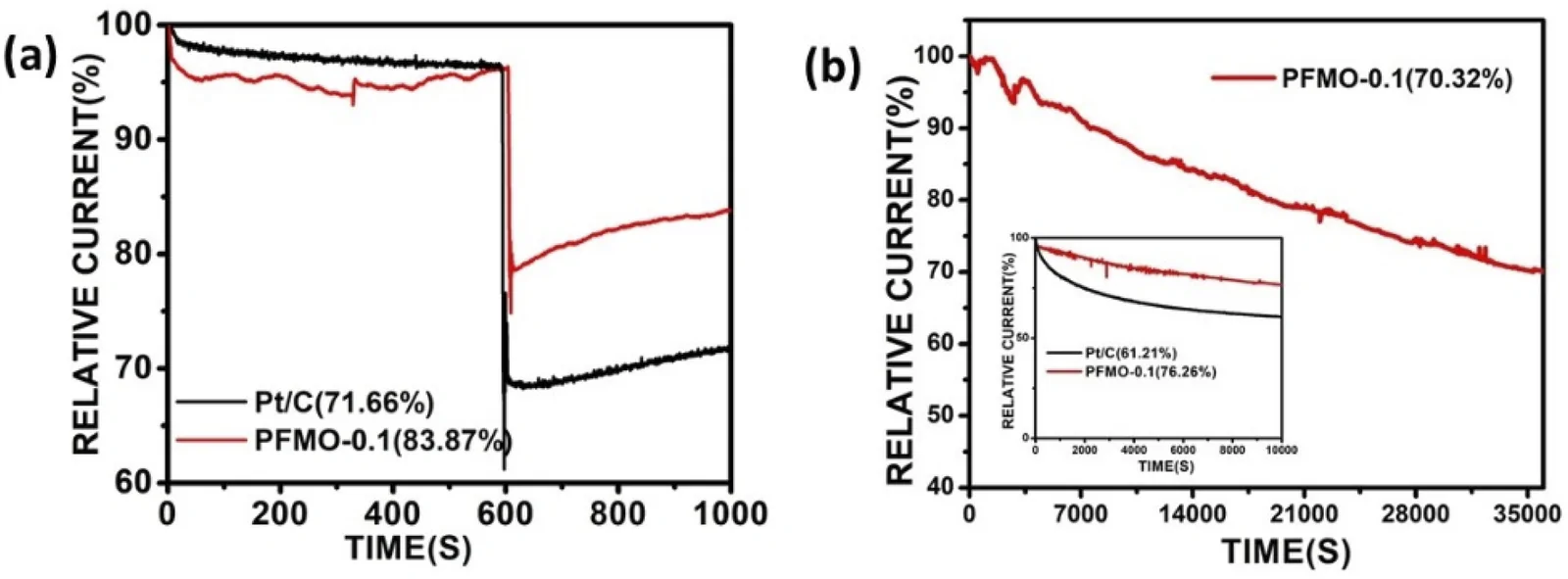
(a) Methanol tolerance test and (b) chronoamperometry test were conducted at −0.35 V *vs.* RHE of PFMO-0.1 at 1600 RPM for 36000 s (10 h) in O_2_-saturated 0.1 M KOH solution, inserting chronoamperometry Pt/C and PFMO-0.1 for 10000 s.

To evaluate methanol tolerance, the ORR current of PFMO-0.1 and a commercial Pt/C catalyst was compared after adding 1.21 mL of methanol. This is due to the addition of 3 M methanol in proportions with KOH (Fig. 13(a)).^61^ The chronoamperometric response of the PFMO-0.1 specimen returned to baseline after approximately 600 seconds post-methanol injection, indicating that the synthesized catalyst remains unaffected by methanol crossover. After 600 seconds, the ORR current for PFMO-0.1 showed only a minor change (retaining 83.87%), whereas the Pt/C catalyst's ORR current dropped significantly (retaining 71.66%), demonstrating PFMO-0.1's superior catalytic ability and nullifying the methanol crossover.

Furthermore, the longevity of PFMO-0.1, analysed using chronoamperometry (Fig. 13(b)), was found to be superior, with PFMO-0.1 maintaining 76.26% of its initial values after 10 000 seconds in an O_2_-saturated 0.1 M KOH electrolyte, outperforming the Pt/C catalyst, which retained only 61.26% of its reduction current over the same period, clearly highlighting the potential of PFMO-0.1 in DMFC. After that, we also take chrono-amperometry for 36 000 s of PFMO-0.1, which retains stability 70.32% means catalyst has great stability.^62^

**Fig. 14:**
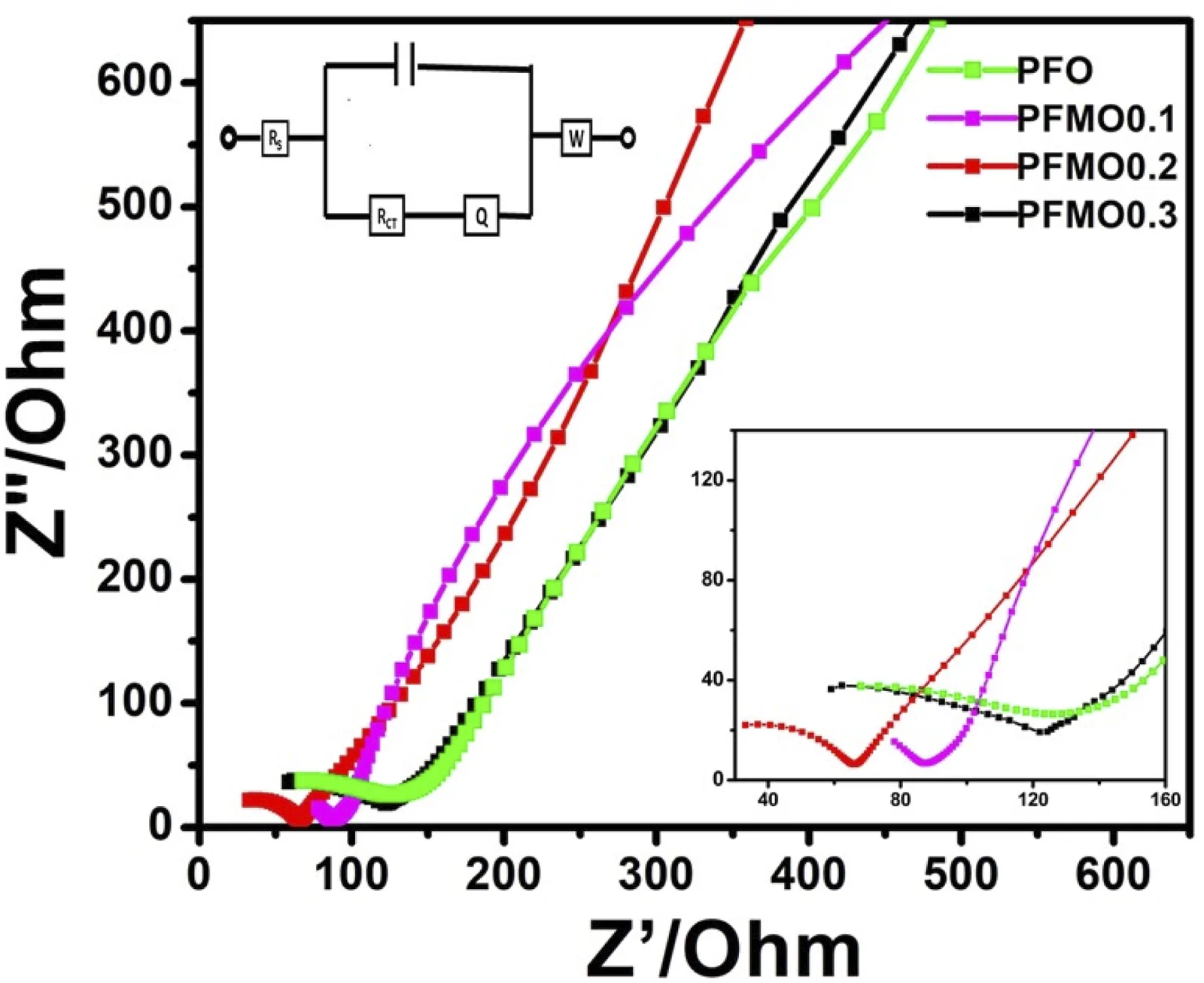
Electrochemical impedance spectroscopy (EIS) Nyquist plot for the synthesized PFMO perovskite.

The interfacial charge transfer processes of the synthesized electrocatalyst and electrolyte were studied by Electrochemical Impedance Spectroscopy (EIS), and the Nyquist plot was used to investigate the diffusion and charge transfer at the electrode–electrolyte interface. The Nyquist plot in Fig. 14 shows a semi-circular part that represents the interfacial charge transfer resistance (*R*_CT_) followed by a linear part that represents the diffusion of the electrolyte at the surface of the electrode; the plot is composed of an equivalent circuit that includes (*R*_CT_), solution resistance (*R*_S_), constant phase element (*C*_PE_), and Warburg impedance (*W*). The calculated results for PFMO catalysts are 43.69 (*R*_CT_) and 10.42 (*R*_S_), whereas for PFO catalysts, they are 66.84 (*R*_CT_) and 26.44 (*R*_S_), respectively.^63^ Finally, we compare the ORR activity of A/B-site doped perovskites by considering *E*_on_, the electron transport number (*n*), and *E*_1/2_. In the stable, it is clearly visible that the electrocatalyst properties of the synthesised perovskites are less or more than those of the other catalysts present in the table. So here we confirmed that our material has moderate properties towards ORR (Table 4).

**Table 4:** Various types of perovskites and their effect on ORR

Electrocatalyst	Method of synthesis	*E*_onset_(V *vs.* RHE)	*n*	*E*_1/2_ (V *vs.* RHE)	Ref. this work
PrFe_0.9_Mn_0.1_O_3_	Sol–gel	0.76	3.85	0.59	My work
PrFe_0.8_Mn_0.2_O_3_	Sol–gel	0.73	3.75	0.54	
PrFe_0.7_Mn_0.3_O_3_	Sol–gel	0.7	3.77	0.50	
LaNi_0.85_Mg_0.15_O_3_	Electrospinning	0.82	3.4		5
PrNi_*x*_Co_1−*x*_O_3_-graphene composite	Sol–gel	∼0.68	3.5		20
La_0.95_Fe_0.05_O_3_	Sol–gel	∼0.63	3.96		24
LaMn_0.1_Fe_0.9_O_3_	Sol–gel	∼0.64	3.87		29
Vulcan carbon with SrMn_0.9_Co_0.1_O_3_(700)	Sol–gel	0.88	3.85	0.70	64
GdCoO_3_/NC composite (400)	High-energy ball milling	0.93	3.7	0.82	65
Ag/La_0.6_Sr_0.4_CoO_3−*δ*_ composite	Sol–gel then electroless	0.72	∼4	0.62	66
La_0.8_Sr_0.2_Mn_0.7_Co_0.3_O_3_	Sol–gel	0.77	3.86		67
S doped La_0.8_Sr_0.2_(CrMnFeCoNi)O_3_(750)	Coprecipitation	0.80	3.87	0.731	68
La_0.7_Sr_0.3_Co_0.2_Mn_0.2_Ni_0.2_Fe_0.2_Al_0.2_O_3_ (HEP)	Sol–gel	0.761	∼4	0.605	69
LaNi_0.5_Co_0.5_O_3_/Vulcan (700)	Sol–gel	0.79	3.11		70
La_0.5_Sr_0.5_Fe_0.8_Cu_0.2_O_3_(800)	Sol–gel	0.572	∼4		71
La_0.5_Sr_0.5_Fe_0.8_Zn_0.2_O_3_(800)	Sol–gel	0.616	∼4		71
LaMn_0.3_Co_0.7_O_3_	Electrospinning	0.72		0.59	72

## Conclusion

A series of manganese-substituted PrFeO3 at the B-site (PrFe_1−*x*_Mn_*x*_O3, *x* = 0.1, 0.2, and 0.3) catalysts, named PFMO-0.1, PFMO-0.2 and PFMO-0.3, respectively, were prepared *via* the sol–gel method and characterized by XRD, SEM, TEM, EDX and XPS. The XRD results revealed that the prepared PFMO-0.1 catalyst has an orthorhombic structure with a length of 4.14 nm and an interplanar distance of 2.758 Å of all the prepared electrocatalysts, PFMO-0.1 was found to be the most efficient ORR electrocatalyst with an onset potential of 0.76 V *vs.* RHE, Tafel slope of 84 mV dec^−1^ and *E*_1/2_ = 0.59 V. The chronoamperometric analysis also revealed that the PFMO-0.1 electrocatalyst is extremely stable and highly tolerant to methanol. As a result, PFMO-0.1 was determined as the best electrocatalyst among all the obtained perovskite materials for ORR.

## Author contributions

P. Parida: writing – original draft, methodology, investigation. B. B. Nayak: methodology, formal analysis A. R. Panda: formal analysis, data curation. T. Hati: review and editing, S. Samanta: investigation. P. Parhi: writing – review and editing, conceptualization.

## Conflicts of interest

The Authors declare that they have no known competing financial interests or personal relationships that could have appeared to influence the work reported in this paper.

## Data Availability

All experimental details, synthesis procedures, and characterization data required for reproducibility have been provided in the main manuscript file.
